# A Self-Oscillator Based on Liquid Crystal Elastomer Fiber Under Constant Voltage

**DOI:** 10.3390/polym16223192

**Published:** 2024-11-17

**Authors:** Dali Ge, Xin Liu, Qingrui Hong, Haiyi Liang

**Affiliations:** 1 School of Civil Engineering, Anhui Jianzhu University, Hefei 230601, China; 2 IAT-Chungu Joint Laboratory for Additive Manufacturing, Institute of Advanced Technology, University of Science and Technology of China, Hefei 241200, China; 3 CAS Key Laboratory of Mechanical Behavior and Design of Materials, Department of Modern Mechanics, University of Science and Technology of China, Hefei 230026, China

**Keywords:** self-oscillation, electrothermal-responsive, liquid crystal elastomer, fiber, self-controlled dynamic circuit

## Abstract

Self-oscillation is the phenomenon in which a system generates spontaneous, consistent periodic motion in response to a steady external stimulus, making it highly suitable for applications in soft robotics, motors, and mechatronic devices. In this paper, we present a self-oscillator based on liquid crystal elastomer (LCE) fiber under constant voltage. The system primarily consists of an LCE–liquid metal (LCE-LM) composite fiber, a metal mass sphere, and a straight rod featuring both conductive and insulating segments. Building upon an established dynamic LCE model, we derive the governing dynamic equations. Numerical calculations reveal two distinct motion regimes: a static regime and a self-oscillation regime. Furthermore, we provide the temporal behavior curves of electrothermal-induced contraction and tensile force, the phase trajectories variation curves of the equivalent driving force and damping force. These detailed studies elucidate that self-oscillation results from the contraction of the electrothermal-responsive LCE-LM fiber when the circuit is activated, with continuous periodic motion being sustained through the interplay between the metal mass sphere and a self-controlled dynamic circuit. We also investigate the threshold conditions necessary for initiating self-oscillation, as well as the key system parameters that influence its frequency and amplitude. Our self-oscillator demonstrates improved stability by reducing the effects of gravity and other disturbances. Additionally, the curved trajectory of the mass sphere can be achieved by replacing the straight rod with a curved one, resulting in a more flexible and easily controllable structure. Given these characteristics, a self-oscillator system based on LCE-LM fiber may be ideal for creating monitoring and warning devices, dynamic circuit systems, and for integrating actuators and controllers.

## 1. Introduction

Self-oscillation is the phenomenon where a system, in response to a constant external stimulus or input, produces spontaneous and regular periodic motion due to an internal nonlinear feedback mechanism [1]. Firstly, self-oscillation greatly eases the burden on a supplementary system regulator, since it relies on a steady external stimulus instead of requiring a periodic one for its oscillatory behavior [2,3]. Secondly, the self-oscillation system actively absorbs energy from a steady external environment to sustain its periodic motion. In addition, self-oscillation exhibits high robustness, and its oscillation frequency and period are mainly controlled by the intrinsic properties of the system [4]. Consequently, due to these advantageous characteristics, self-oscillation systems are emerging as promising candidates for various applications, including autonomous robotics [5,6,7,8], energy-absorbing devices [9,10], mechano-logistic devices [11,12,13], biomimetic designs [14], and so on.

Recently, researchers have developed a diverse range of self-oscillation systems through utilizing stimulus-responsive materials, and this has not only enhanced our comprehension of the mechanisms behind self-oscillation but has also paved the way for new practical applications. By utilizing hydrogels [15,16], polydimethylsiloxane [17], ethylene-co-vinyl acetate [18], crystals [19], and liquid crystal elastomers (LCEs) [6,20,21], researchers designed various fundamental self-sustaining motion patterns, such as vibration [22,23,24], bending [25,26,27,28], rolling [7,29,30,31], torsion [32,33], stretching and shrinking [34,35], swimming [8], swinging [36,37], buckling [38,39,40,41,42], jumping [43,44,45,46,47], rotation [48,49,50,51], galloping [52], eversion or inversion [53,54], snap-through [55], and even the synchronized motion of several coupled self-oscillators [56]. Self-oscillation motions are typically regulated by inherent instabilities or feedback mechanisms within their structure or dynamics. These mechanisms include phase transitions in molecular assembly [57], self-shadowing effects [58,59], mechanical zero-elastic-energy mode [53], the Belousov–Zhabotinsky reaction [60], chemo-mechanical coupling between the large deformation and reaction–diffusion processes [20], and photothermal solvent evaporation [61].

LCEs represent a notable class of active materials characterized by exceptional stimulus-responsive properties, effectively blending the anisotropic nature of liquid crystals with the rubbery elasticity of polymer networks [62,63]. When subjected to environmental stimuli [64,65,66,67,68,69,70,71,72], the LCEs undergo a transition from the liquid crystal phase to the isotropic phase, resulting in rapid and significant shape morphing [73]. Owing to their advantageous properties, such as mild stimulus requirements, large deformations, and anisotropic contraction, LCEs are highly esteemed as excellent candidates for fabricating self-oscillating systems. Most previously reported LCE materials have been engineered to respond to heat or light stimuli. However, certain technical challenges, such as the requirement for complex equipment and limited light penetration, pose significant obstacles in regard to their practical use. In contrast, electrical energy being the most prevalent energy source in society offers several advantages, including convenience, cleanliness, low cost, and high adjustability. This makes it the preferred driving force for modern devices. The ability to directly convert electrical energy into mechanical work is crucial for the practical application of electrothermal-responsive LCE materials [74]. In this regard, electrothermal-responsive self-oscillation systems based on LCEs have been demonstrated [33,75,76,77,78].

To enhance the applicability of self-oscillation systems and facilitate the design and creation of more functional soft actuators, it is essential to develop more diverse self-sustaining systems. In this article, we propose a self-oscillator based on LCEs under constant voltage. The system comprises an electrothermal-responsive LCE–liquid metal (LCE-LM) composite fiber, a metal mass sphere, a straight rod featuring both a conductive segment and an insulating segment, conductive wire, and a direct current supply. It produces self-oscillation by causing the LCE-LM fiber to contract when the circuit is activated, and the continuous periodic motion is sustained through the interplay between the metal mass sphere and a self-controlled dynamic circuit. This self-oscillator primarily transforms electrical energy into kinetic energy instead of increasing gravitational potential energy, fulfilling the kinetic energy requirements in many practical applications. Additionally, external factors like initial displacement and velocity need to surpass a specific threshold to initiate self-oscillation, which then leads to a continuous consumption of electrical energy. Unlike self-oscillating flexible circuits [79], our self-oscillator exhibits better stability because the mass sphere slides along a straight rod, reducing the effects of gravity and other disturbances. Additionally, the curved trajectory of the mass sphere can potentially be achieved by replacing the straight rod with a curved one, resulting in a more flexible and easily controllable structure. Given these characteristics, the proposed self-oscillator system based on LCE-LM fibers may be ideal for creating monitoring and warning devices, dynamic circuit systems, and for integrating actuators and controllers.

The remaining framework of this paper is outlined as follows: In Section 2, we establish the theoretical model and dynamic governing equations for the self-oscillator based on LCE-LM fiber under constant voltage. In Section 3, we introduce two regimes of the self-oscillator under constant voltage: the static regime and the self-oscillation regime. Additionally, a comprehensive explanation of the mechanism underlying the emergence of self-oscillation is presented. In Section 4, the threshold for triggering self-oscillation and the influences of system parameters on the self-oscillation amplitude and frequency are studied. Finally, Section 5 provides a brief summary of the paper’s findings.

## 2. Theoretical Framework

In this section, a theoretical model is formulated for a self-oscillator based on LCE-LM composite fiber under constant voltage, including the fundamental equations for the motion of the metal sphere, the dynamics model of the electrothermal temperature, nondimensionalization, and the solution method.

### 2.1. Dynamics of Self-Oscillator

**Figure 1:**
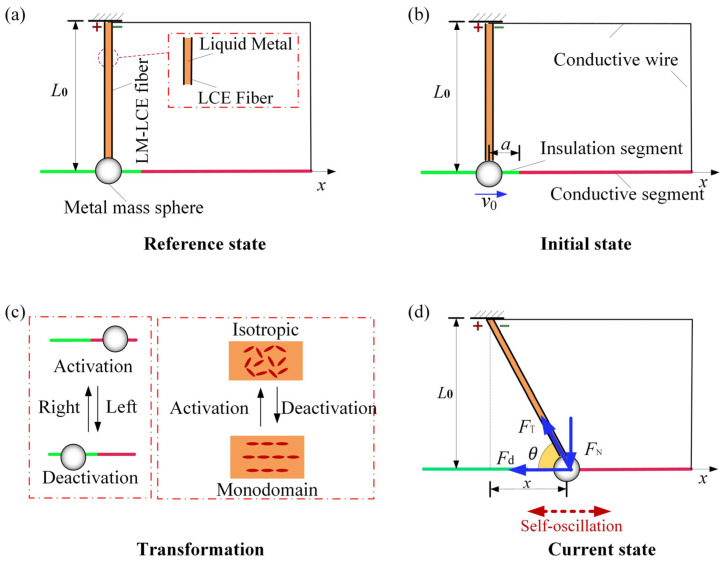
Schematic of a self-oscillating LCE-LM fiber-oscillator system under constant voltage. (**a**) Reference state, (**b**) Initial state, (**c**) Transformation, (**d**) Current state.

Figure 1 illustrates a self-oscillating LCE-LM fiber-oscillator system under constant voltage composed of an electrothermal-responsive LCE-LM fiber with an initial length $L_{0}$, metal mass sphere with a hole, a straight rod featuring both a conductive segment and an insulating segment, conductive wire, and a direct current supply. The uniaxially oriented LCE fiber is produced by stretching. Through first plasma treatment and then roll coating, the electrothermal-responsive LCE-LM composite fiber can be fabricated [75]. Figure 1a shows the reference state of the system. The direct current supply is placed on the fixed end. One end of the LCE-LM fiber is clamped and connected to the direct current supply; the other end is connected to the metal sphere with mass *m*. The straight rod passes through the hole of the metal sphere so that the metal sphere can move freely on the straight rod. The insulating segment is on the left side of the straight rod, indicated in green, while the conductive segment is on the right side of the straight rod, indicated in red. It is noted that, in the initial state, the LCE-LM is straight and stress-free, as shown in Figure 1b, and the junction position of the conductive and insulating segments is at distance a from the initial position of the metal sphere. The right end of the conductive segment is linked to the direct current supply through the conductive wire. The electrothermal-responsive LCE-LM fiber, metal sphere, the straight rod, conductive wire, and direct current supply form a complete circuit. Whether the circuit is activated or inactivated depends on whether the conductive metal sphere contacts the conductive segment or the insulating segment of the straight rod. As the metal sphere oscillates, switching the circuit between activation and deactivation, the thermally responsive liquid crystal molecules in the LCE fiber transition between a monodomain configuration and an isotropic phase. This process causes the LCE fiber to contract and elongate along its length, as depicted in Figure 1c.

In the initial state, the metal sphere is in contact with the insulating segment of the straight rod and the circuit is activated. By giving the metal sphere an initial rightward velocity $v_{0}$, it moves along the straight rod and then makes contact with the conductive segment, which activates the circuit and causes the LCE-LM fiber to undergo a gradually increasing electrothermally induced contraction due to electro-joule heating. Therefore, under the joint effect of increasing the LCE fiber length and electrothermally induced contraction, the tensile force $F_{T}\left(t\right)\;$progressively increases. This causes the metal sphere to gradually decelerate to zero speed and subsequently initiate leftward movement. When the metal sphere reaches the insulating segment of the straight rod, the circuit is activated and the electrothermally induced contraction gradually recovers. As the LCE fiber elongates, its tensile force $F_{T}\left(t\right)$ progressively increases, causing the metal sphere to gradually decelerate and subsequently move back to rightward. Therefore, the motion of the metal sphere is coupled with the oscillation between the circuit’s activated and inactivated states, resulting in periodic oscillation of the metal sphere when supplied with a direct current.

To describe the motion of the metal sphere, a displacement coordinate system is established with the coordinate origin O set at the initial position of the metal sphere, and the coordinate axis is oriented along the right direction of the straight rod. The instantaneous displacement of the metal sphere during its movement at time *t* can be expressed as $x\left(t\right)$, and the current length of the LCE-LM fiber can be recorded as *L*$\left(t\right)$. According to Figure 1d, the relationship between *L*$\left(t\right)$ and $x\left(t\right)$ can be obtained as follows: *L*$\left(t\right)=\sqrt{x^{2}\left(t\right)+L_{0}^{2}}.$ During the motion of the metal sphere, we assume that the friction between the metal sphere and the straight rod is negligible. Under this assumption, the metal sphere experiences a tensile force $F_{T}\left(t\right)$ along the LCE-LM fiber, along with a damping force $F_{d}\left(t\right)$ and a contact force $F_{N}\left(t\right)$ from the straight rod, as shown in Figure 1d. To streamline our analysis, we assume that the damping force $F_{d}\left(t\right)$ acting on the metal sphere is directly related to its velocity $\dot{x}\left(t\right)$. Consequently, the governing formula for the metal sphere can be expressed as:

(1) $$m\ddot{x}\left(t\right)=-F\left(t\right)-\beta \dot{x}\left(t\right),$$

where $\ddot{x}\left(t\right)$ represents the acceleration of the metal sphere and β denotes the damping coefficient. The initial condition is stated as follows:

(2) $$x\left(t\right)=0,\dot{x}\left(t\right)=v_{0}\;at\;t=0.$$

It is important to note that the equivalent driving force $F\left(t\right)$ in Equation (1) represents the component of the tensile force $F_{T}\left(t\right)$ along the direction of the metal sphere’s motion. It is determined by the combined effect of the $F_{T}\left(t\right)\;$and the cosine of the angle θ between the fiber and the straight rod. Therefore, $F\left(t\right)\;$can be written as:

(3) $$F\left(t\right)=F_{T}\left(t\right)\cos\theta .$$

The cos⁡θ can be expressed in terms of geometric relationships as follows:

(4) $$\cos\theta =\frac{x\left(t\right)}{\sqrt{x^{2}\left(t\right)+L_{0}^{2}}}.$$

According to Equations (3) and (4), Equation (1) can be rewritten as:

(5) $$m\ddot{x}(t)=-F_{T}(t)\frac{x\left(t\right)}{\sqrt{x^{2}\left(t\right)+L_{0}^{2}}}-\beta \dot{x}\left(t\right),$$

In the case of small deformations of the LCE fiber, we assume that the tensile force $F_{T}(t)$ is linearly connected to the elastic strain $\varepsilon_{e}\left(t\right)$. This can be expressed as:

(6) $$F_{T}(t)=kL_{0}\varepsilon_{e}\left(t\right),$$

where k is the elastic coefficient of the LCE-LM fiber. The elastic strain $\varepsilon_{e}\left(t\right)$ under small deformation can be considered a linear combination of the total strain $\varepsilon_{tot}\left(t\right)$ and the electrothermally induced contraction strain$\;\varepsilon \left(t\right)$, i.e., $\varepsilon_{e}\left(t\right)=\varepsilon_{tot}\left(t\right)-\varepsilon \left(t\right)$. Therefore, the tensile force $F_{T}(t)$ can be written as:

(7) $$F_{T}(t)=kL_{0}(\varepsilon_{tot}\left(t\right)-\varepsilon \left(t\right)),$$

where $\varepsilon_{tot}\left(t\right)$ can be expressed in terms of geometric relationships as follows:

(8) $$\varepsilon_{tot}\left(t\right)=\left(\sqrt{x^{2}\left(t\right)+{L_{0}}^{2}}-L_{0}\right)/L_{0},$$

According to Equations (7) and (8), the tensile force $F_{T}(t)$ of Equation (6) can be rewritten as:

(9) $$F_{T}\left(t\right)=k\left[\sqrt{x^{2}\left(t\right)+{L_{0}}^{2}}-L_{0}-L_{0}\varepsilon \left(t\right)\right].$$

The electrothermally induced contraction strain $\varepsilon \left(t\right)\;$is presumed to have a linear relationship with the temperature difference $T\left(t\right)$ of the LCE-LM fiber [75]. This can be expressed as follows:

(10) $$\varepsilon \left(t\right)=-C_{0}T\left(t\right),$$

where $C_{0}\;$is the thermal contraction coefficient.

To calculate the tensile force of the LCE fiber, its temperature difference $T\left(t\right)$ is calculated in the following section.

### 2.2. Dynamics Model of Electrothermal Temperature

To establish the temperature difference $T\left(t\right)$ in Equation (10), we assume that heat exchange occurs rapidly enough for the temperature within the electrothermally responsive LCE-LM composite fiber to remain uniform. Consequently, the temperature within the LCE-LM composite fiber is considered to be uniformly distributed over time. Under these conditions, the heat energy converted from electrical energy during conduction can be calculated using Joule’s law of heating [75]. Assuming that the LCE-LM composite fiber is the only resistive component in the circuit system, the electrothermal flow Q, which represents the thermal energy converted from electrical energy per unit time, can be expressed in terms of current I and resistance R of the LCE-LM fiber, i.e.,

(11) $$Q=I^{2}R,$$

Since heat always transfers from an object at a higher temperature to one at a lower temperature, we can express the temperature difference $T\left(t\right)$ during the heat loss $Q_{l}$ in the context of LCE-fiber heat transfer as follows:

(12) $$\frac{dT\left(t\right)}{dt}=\frac{Q-Q_{l}}{\rho_{C}},$$

where $\rho_{C}$ represents the specific heat capacity. The heat lost $Q_{l}$ can be assumed to have a linear relationship with the LCE-LM composite fiber’s relative temperature compared to the ambient temperature, expressed as:

(13) $$Q_{l}=k_{c}T\left(t\right),$$

where $k_{c}$ denotes the heat transfer coefficient. Equation (13) can be rewritten as:

(14) $$\frac{dT\left(t\right)}{dt}=\frac{Q-k_{c}T\left(t\right)}{\rho_{C}},$$

Here, $T_{max}=\frac{Q}{k_{c}}$ defines the maximum temperature difference for the LCE-LM composite fiber when a constant electrical potential is applied and $\tau =\frac{\rho_{C}}{k_{c}}$ represents the thermal relaxation time, reflecting the rate of heat exchange between the LCE-LM composite fiber and its surroundings. Thus, Equation (14) can be further expressed as:

(15) $$\frac{dT\left(t\right)}{dt}=\frac{T_{max}-T\left(t\right)}{\tau },$$

It is important to note that, when the circuit is inactivated, we can set the electrothermal flow Q=0, which allows us to yield Equation (14):

(16) $$\frac{dT\left(t\right)}{dt}=\frac{-k_{c}T\left(t\right)}{\rho_{C}}.$$

### 2.3. Nondimensionalization

To simplify the discussion, we introduce the dimensionless quantities in the following manner: $\bar{t}=t/\tau$, $\bar{a}=a/L_{0},\;\bar{\beta }=\beta \tau /m,\;$$\bar{k}=k\tau^{2}/m$, ${\bar{F}}_{T}=F_{T}\tau^{2}/\left(mL_{0}\right),\;{\bar{F}}_{d}=F_{d}\tau^{2}/\left(mL_{0}\right),\;\bar{x}\left(t\right)=x/L_{0},$ $\bar{\dot{x}}=\dot{x}\tau /L_{0}$, $\bar{\ddot{x}}=\ddot{x}\tau^{2}/L_{0}$, ${\bar{C}}_{0}=C_{0}T_{max}$, $\bar{T}=T/T_{\mathrm{ext}}$, $\bar{Q}=Q/k_{c}T_{\mathrm{ext}}$, ${\bar{x}}_{0}=x_{0}/L_{0},\;{\bar{v}}_{0}=v_{0}\tau /L_{0}$. Therefore, the governing equations in Equations (5), (9) and (10) of the metal sphere are nondimensionalized, as follows:

(17) $$\bar{\ddot{x}}\left(\bar{t}\right)=-{\bar{F}}_{T}\left(t\right)\frac{\bar{\bar{x}}\left(t\right)}{\sqrt{{\bar{x}}^{2}\left(\bar{t}\right)+1}}-\bar{\beta }\bar{\dot{x}}\left(\bar{t}\right),$$

(18) $$\bar{x}\left(\bar{t}\right)={\bar{x}}_{0},\;\bar{\dot{x}}(t)={\bar{v}}_{0}\;at\;\bar{t}=0,$$

where ${\bar{F}}_{T}\left(t\right)$ is

(19) $${\bar{F}}_{T}\left(t\right)=\bar{k}\left[\sqrt{{\bar{x}}^{2}\left(\bar{t}\right)+1}-1-\varepsilon \left(\bar{t}\right)\right],$$

with

(20) $$\varepsilon \left(\bar{t}\right)=-{\bar{C}}_{0}\bar{T}\left(\bar{t}\right).$$

The dimensionless temperature difference in Equations (15) and (16) can be determined as

(21) $$\frac{d\bar{T}\left(\bar{t}\right)}{d\bar{t}}=\bar{Q}-\bar{T}\left(\bar{t}\right)\;for\;the\;active\;state,$$

and

(22) $$\frac{d\bar{T}\left(\bar{t}\right)}{d\bar{t}}=\bar{Q}-\bar{T}\left(\bar{t}\right)\;for\;the\;inactive\;state.$$

### 2.4. Solution Method

Equations (17), (21) and (22) govern the self-oscillator based on electrothermal-responsive LCE fiber under constant voltage, where the time-dependent temperature difference is coupled with the displacement of the metal sphere. Hereon, to solve these complex variable coefficient differential equations, the Runge–Kutta method is utilized and numerical calculation is carried out with Matlab 2023a software. The equations utilized in the fourth-order Runge–Kutta method are presented below:

(23) $$x\left(\bar{t}+h\right)=x\left(\bar{t}\right)+\frac{1}{6}\left(L_{1}+2L_{2}+3L_{3}+4L_{4}\right),$$

where $L_{i}\left(i=1,2,3,4\right)$ is expressed as:

(24) $$\left\{\begin{array}{c} L_{1}=hf\left(\bar{t},x\right)\\ L_{2}=hf\left(\bar{t}+\frac{1}{2}h,x+\frac{1}{2}L_{1}\right)\\ L_{3}=hf\left(\bar{t}+\frac{1}{2}h,x+\frac{1}{2}L_{2}\right)\\ L_{4}=hf\left(\bar{t}+h,x+L_{3}\right) \end{array}\right.,$$

where h is the time step and $f\left(\bar{t},x\right)\;$takes a two-dimensional vector form as below:

(25) $$\left[\begin{array}{c} \bar{\dot{x}}\\ -{\bar{F}}_{T}\left(t\right)\frac{\bar{\bar{x}}\left(t\right)}{\sqrt{{\bar{x}}^{2}\left(\bar{t}\right)+1}}-\bar{\beta }\bar{\dot{x}}\left(\bar{t}\right) \end{array}\right]$$

Ultimately, the dynamic response of the displacement $x\left(\bar{t}\right)$ and velocity $\bar{\dot{x}}\left(\bar{t}\right)\;$for the oscillating behavior over time can be obtained by iterative analysis. In the computation, after performing the stability analysis and verifying the results numerically, we set the time step h=0.001. It is worth mentioning that, based on the displacement of the metal sphere ${\bar{x}}_{n}$ and the electrothermally induced contraction $\varepsilon_{n}$ at the current moment, we can determine the current tensile force ${\bar{F}}_{T(n)}$ according to Equation (19). Then, the displacement ${\bar{x}}_{n+1}$ at the next moment is further determined from Equation (16). The temperature difference ${\bar{T}}_{n+1}$ at the next moment is calculated from Equation (21) or (22). It is noted that the circuit is activated for ${\bar{x}}_{n+1}>{\bar{a}}_{0}$; otherwise, the circuit is inactivated. Furthermore, the contraction strain $\varepsilon_{n}$ is calculated from Equation (20). Next, using the electrothermally induced contraction strain $\varepsilon_{n+1}$ and the displacement ${\bar{x}}_{n+1}\;$at the next moment, we can further calculate the tensile force ${\bar{F}}_{T(n+1)}$ at that moment according to Equation (19). By conducting successive computations, we can derive the temporal behavior of the self-oscillator based on the electrothermal-responsive LCE fiber for given parameters ${\bar{x}}_{0},\;{\bar{v}}_{0},\;\bar{\beta },\;\bar{k},\;{\bar{C}}_{0},\;\bar{a},$ and $\bar{Q}$, allowing for further investigation into the effects of various parameters on its dynamic behavior.

## 3. Two Motion Regimes and the Mechanism of Self-Oscillation

In this section, we begin by presenting two typical motion regimes of the self-oscillator based on the electrothermal-responsive LCE fiber, specifically the static regime and the self-oscillation regime, using the governing Equations (17)–(22). Following this, we provide a detailed explanation of the mechanisms underlying self-oscillation.

### 3.1. Two Motion Regimes

This article aims to investigate the cyclic behavior of a self-oscillator that employs an electrothermal-responsive LCE fiber, necessitating an estimation of the relevant dimensionless parameters. Table 1 summarizes the system parameters obtained from earlier experiments [67,75]. By referencing the typical dimensionless parameters outlined in Table 2, we assess the dynamic behavior of the self-oscillator under constant voltage conditions.

**Figure 2:**
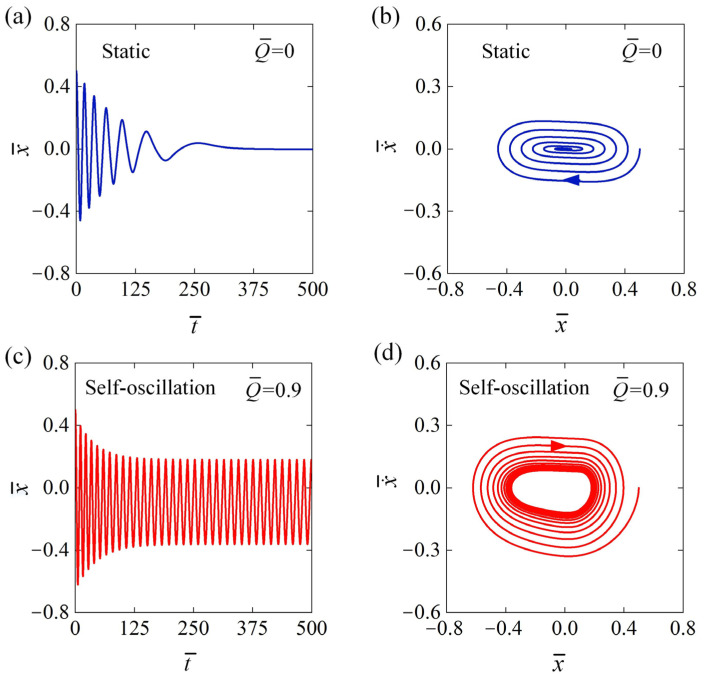
Two distinct motion regimes: the static regime and the self-oscillation regime. (**a**) Displacement temporal behavior with $\bar{Q}=0$; (**b**) phase trajectory with $\bar{Q}=0$; (**c**) displacement temporal behavior with $\bar{Q}=0.9;$ and (**d**) phase trajectory with $\bar{Q}=0.9$. Other parameters are ${\bar{x}}_{0}=0.5,\;{\bar{v}}_{0}=0,\;\bar{\beta }=0.03,\;\bar{k}=2,\;{\bar{C}}_{0}=0.3,\;and\;\bar{a}=0.1.$ When the oscillator is subjected to constant voltage, it exhibits two primary motion regimes: the static regime and the self-oscillation regime.

Figure 2 presents the temporal behavior of displacement and the phase trajectory plots for the two motion regimes of the self-oscillator utilizing LCE fiber while maintaining a constant voltage. In our numerical simulations, we employed the following parameters: ${\bar{x}}_{0}=0.5,\;{\bar{v}}_{0}=0,\;\bar{\beta }=0.03,\;\bar{k}=2,\;{\bar{C}}_{0}=0.3,\;\bar{a}=0.1.$ Figure 2a,b depict the static regime in the absence of electrical excitation, where $\bar{Q}=0$. When the metal sphere is initially positioned at a non-static equilibrium point, it begins to oscillate back and forth. However, due to damping, both its amplitude and velocity gradually decline over time until it comes to rest at the equilibrium position, a state referred to as the static regime. In contrast, Figure 2c,d show the self-oscillation regime under electrothermal excitation with $\bar{Q}=0.9$. The metal sphere initially oscillates back and forth, with its amplitude and velocity gradually decreasing over time until they stabilize. Under constant voltage, the metal sphere ultimately exhibits continuous and stable periodic motion. The detailed explanation of the self-oscillation mechanism can be found in Section 3.2.

### 3.2. Mechanisms of Self-Oscillation

**Figure 3:**
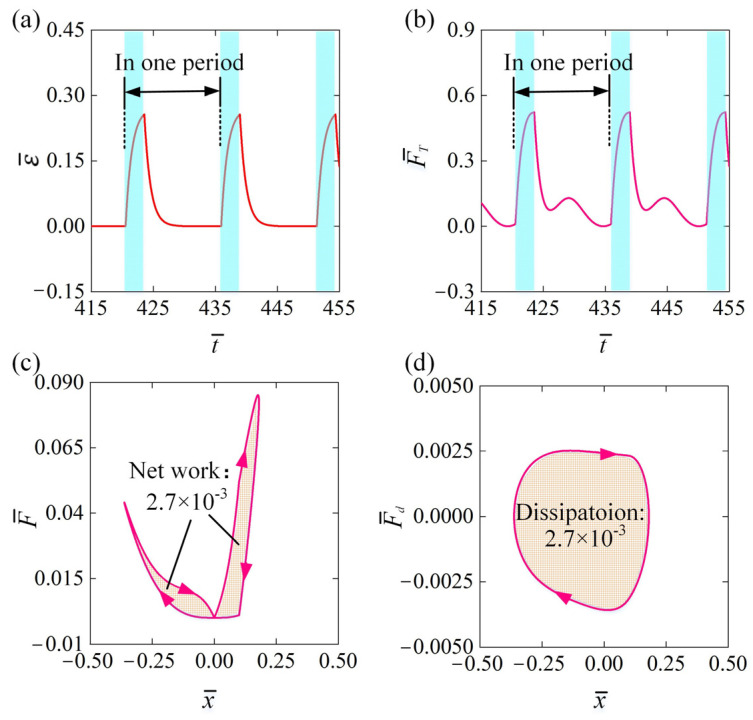
The mechanism of a self-oscillator based on LCE fiber under constant voltage. The parameters were defined for the scenarios presented in Figure 2c,d. (**a**) The temporal behavior of the electrothermally induced contraction. (**b**) The temporal behavior of the tensile force. (**c**) The relationship between the equivalent driving force and displacement. (**d**) The relationship of the damping force with displacement. The positive net work performed by the tensile force compensates for damping losses, enabling sustained self-oscillation.

To examine how the self-oscillator based on LCE fiber operates under constant voltage, Figure 3 showcases the progression of various essential parameters of the LCE-LM fiber for the scenarios presented in Figure 2c,d. Figure 3a illustrates the time-dependent periodic changes in electrothermally induced contraction, with the blue-shaded region representing the activated state of the circuit. As shown in Figure 3a, when the circuit is activated, the electrothermally induced contraction strain gradually increases as the temperature rises. Conversely, while the circuit is switched off, the electrothermally induced contraction strain gradually decreases as the temperature drops. Figure 3b shows the periodic variation in the LCE-LM fiber’s tensile force ${\bar{F}}_{T}\;$over time. When the metal sphere moves toward the right and contacts the conductive segment, ${\bar{F}}_{T}\;$increases due to the elongation of the fiber and the resulting contraction strain. As the metal sphere reaches its furthest right position and begins to return to the initial position, ${\bar{F}}_{T}\;$decreases because the fiber length and contraction strain recover. Once the metal sphere returns to the initial position and continues moving to the left, ${\bar{F}}_{T}\;$increases again due to the further elongation of the fiber. Finally, when the metal sphere reaches its furthest left position and starts moving back to the right, ${\bar{F}}_{T}\;$decreases once more as the fiber length recovers. Thus, the cyclical changes in the electrothermally induced contraction strain result in a corresponding oscillation in the tensile force, as illustrated in Figure 3b.

The variation in the equivalent driving force $\bar{F}\;$can be analogously derived from the changes in the tensile force ${\bar{F}}_{T}\;$and the angle θ. Figure 3c further illustrates the relationship between the equivalent driving force $\bar{F}$ versus the displacement $\bar{x}$ of the metal sphere over one period. The resulting curve forms two clockwise closed loops within a single period of self-oscillation. The area contained within this closed curve signifies the positive net work exerted by the tensile force of the LCE fiber during one complete oscillation cycle, which is numerically estimated to be approximately $2.7\times 10^{-3}$. Similarly, as observed in Figure 3d, the damping force varies with the displacement of the metal sphere over one period, creating a counterclockwise closed curve. The area enclosed by this curve represents the damping dissipation energy, calculated to be $2.7\times 10^{-3}$. The positive net work conducted by the tensile force offsets the damping dissipation caused by the damping force, allowing for the maintenance of a continuous and stable state of self-oscillation.

## 4. Parametric Discussion

In the previously mentioned theoretical model of the self-oscillator, there are seven dimensionless parameters in the system, including ${\bar{x}}_{0},\;{\bar{v}}_{0},\;\bar{\beta },\;\bar{k},\;{\bar{C}}_{0},\;\bar{a}$ and $\bar{Q}$. This section will explore how these system parameters affect the dimensionless threshold value, amplitude A, and frequency f of self-oscillation.

### 4.1. Influence of the Initial Conditions

**Figure 4:**
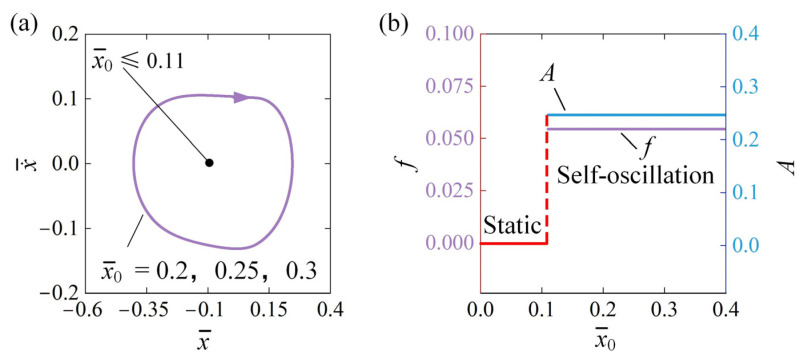
Influence of ${\bar{x}}_{0}\;$on self-oscillation for $\bar{k}=2,\;{\bar{v}}_{0}=0,\;\bar{\beta }=0.03,\;\bar{a}=0.1,\;{\bar{C}}_{0}=0.3,\;and\;\bar{Q}=0.5$. (**a**) Limit cycles; (**b**) effect of ${\bar{x}}_{0}$ on A and f. The oscillator’s motion regime can be controlled by adjusting the initial conditions, while the A and f of the self-oscillator remain unchanged.

Figure 4 illustrates how the initial displacement ${\bar{x}}_{0}$ affects the self-oscillation behavior for $\bar{k}=2,\;{\bar{v}}_{0}=0,\;\bar{\beta }=0.03,\;\bar{a}=0.1,\;{\bar{C}}_{0}=0.3,\;and\;\bar{Q}=0.5.$ Figure 4a shows the cycles corresponding to various initial displacements. There is a threshold initial displacement$\;{\bar{x}}_{0}$ of approximately 0.11 that marks the phase transition between the static regime and the self-oscillation regime. When ${\bar{x}}_{0}\leq 0.11,\;$the system demonstrates a static regime because the energy input is insufficient to offset the damping dissipation. In contrast, self-oscillation of the system can be triggered when ${\bar{x}}_{0}>0.11$. The self-oscillation regime is triggered at${\;\bar{x}}_{0}=0.2$, ${\bar{x}}_{0}=0.25$, and ${\bar{x}}_{0}=0.3$, which have identical limit cycles, as shown in Figure 4a. Figure 4b presents the self-oscillation frequency and amplitude as functions of initial displacement$\;{\bar{x}}_{0}$. It is clear that, as the initial displacement increases, both the f and A stay constant. Importantly, initial velocity ${\bar{v}}_{0}$ transformed into an equivalent initial displacement ${\bar{x}}_{0}$ by utilizing the mechanical energy conversion within the system comprising the LCE fiber and the metal sphere. Thus, it can be concluded that the motion regime of the oscillator can be controlled by adjusting the initial conditions, but the amplitude and frequency of the self-oscillator remain unaffected.

### 4.2. Influence of the Electrothermal Flow

**Figure 5:**
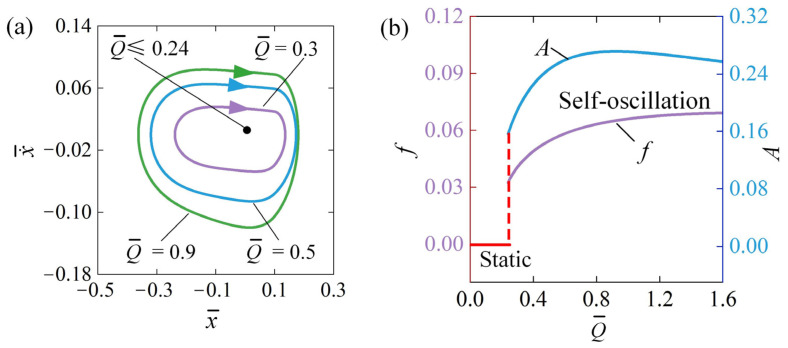
Influence of $\bar{Q}$ on self-oscillation for given values of ${\bar{x}}_{0}=0.5,\;{\bar{v}}_{0}=0,\;\bar{\beta }=0.03,\;\bar{k}=2,\;{\bar{C}}_{0}=0.3,\;and\;\bar{a}=0.1$. (**a**) Limit cycles; (**b**) effect of $\bar{Q}$ on A and f. As the electrothermal flow increases, the f monotonously increases, while A first increases then decreases.

Figure 5 illustrates how the electrothermal flow $\bar{Q}$ influences self-oscillation for ${\bar{x}}_{0}=0.5,\;{\bar{v}}_{0}=0,\;\bar{\beta }=0.03,\;\bar{k}=2,\;{\bar{C}}_{0}=0.3,\;and\;\bar{a}=0.1.$ Figure 5a illustrates the limit cycles associated with various electrothermal flow scenarios. A threshold electrothermal flow $\bar{Q}$ of approximately 0.24 is observed at the phase transition between the static and self-oscillation regimes. In the scenario where $\bar{Q}\leq 0.24,\;$the oscillator operates in a static regime due to insufficient energy input to offset the damping losses. As a result, the system ultimately settles into the static equilibrium position and remains motionless. On the other hand, self-oscillation occurs in the system in the scenario of $\bar{Q}>0.24.$ The limit cycles induced by self-oscillation at $\bar{Q}=0.3$, $\bar{Q}=0.5$, and $\bar{Q}=0.9$ are illustrated in Figure 5a. Figure 5b shows the A and f as functions of electrothermal flow $\bar{Q}$. As the electrothermal flow intensifies, f increases monotonically, while A first increases and then decreases. This phenomenon can be attributed to the increased temperature difference and electrothermal-induced contraction resulting from the rise in electrothermal flow, as described by Equations (20) and (21), facilitating the conversion of electrical energy into mechanical energy, which in turn leads to an increase in amplitude. Simultaneously, the oscillation velocity of the metal sphere also increases, allowing it to complete an oscillation cycle in a shorter amount of time, thereby manifesting as an increase in frequency. However, when the electrothermal flow $\bar{Q}$ is excessively high, causing the velocity to increase too rapidly, the metal sphere may quickly reduce its velocity to zero due to the rapidly increasing tensile force. This, in turn, reduces both the maximum distance traveled on the straight rod and the amplitude of self-oscillation.

### 4.3. Influence of the Contraction Coefficient

**Figure 6:**
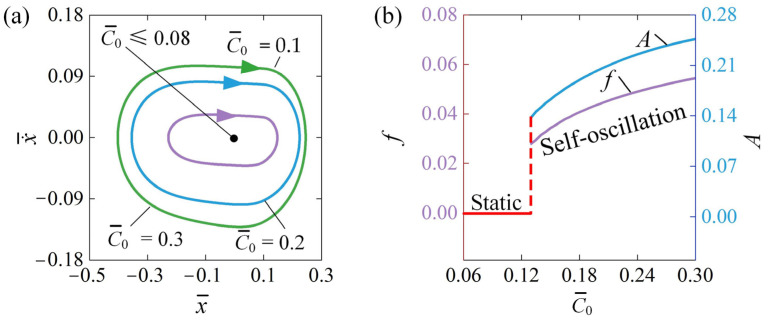
Influence of ${\bar{C}}_{0}$ on self-oscillation for given values of ${\bar{x}}_{0}=0.5,\;{\bar{v}}_{0}=0,\;\bar{\beta }=0.03,\;\bar{a}=0.1,\;\bar{k}=2,\;and\;\bar{Q}=0.5$. (**a**) Limit cycles; (**b**) effect of ${\bar{C}}_{0}$ on A and f. Augmenting the contraction coefficient leads to higher self-oscillation in A and f.

Figure 6 illustrates how the contraction coefficient ${\bar{C}}_{0}$ influences self-oscillation behavior. For this analysis, we set the parameters to ${\bar{x}}_{0}=0.5,\;{\bar{v}}_{0}=0,\;\bar{\beta }=0.03,\;\bar{k}=2,\;\bar{Q}=0.5,\;\mathrm{and}\;\bar{a}=0.1.$ Figure 6a illustrates how the limit cycles of self-oscillation change with various contraction coefficients. Significantly, a threshold contraction coefficient ${\bar{C}}_{0}$ of approximately 0.08 indicates the boundary between static and self-oscillation regimes. When ${\bar{C}}_{0}\leq 0.08,\;$it remains static; however, self-oscillation is initiated when ${\bar{C}}_{0}>0.08$. The limit cycle triggered by the self-oscillation at ${\bar{C}}_{0}=0.1$, ${\bar{C}}_{0}=0.2$, and ${\bar{C}}_{0}=0.3$ is shown in Figure 6a. Figure 6b shows how both frequency and amplitude change as a function of contraction coefficient ${\bar{C}}_{0}$. It is clear that increasing the contraction coefficient leads to a higher self-oscillation frequency and amplitude. Equation (21) indicates that an increase in the contraction coefficient enhances electrothermally induced contraction, which corresponds to a greater tensile force in the LCE fiber. This indicates that enhancing the contraction coefficient can significantly improve the effectiveness of transforming thermal energy into mechanical energy.

### 4.4. Influence of the Damping Coefficient

**Figure 7:**
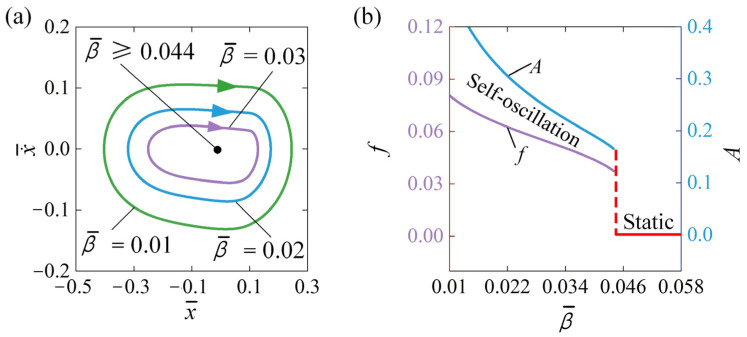
Influence of $\bar{\beta }$ on self-oscillation for given values of ${\bar{x}}_{0}=0.5,\;{\bar{v}}_{0}=0,\;{\bar{C}}_{0}=0.3,\;\bar{a}=0.1,\;\bar{k}=2,\;and\;\bar{Q}=0.5$. (**a**) Limit cycles; (**b**) Effect of $\bar{\beta }$ on A and f. As the damping coefficient increases, both the self-oscillation A and f consistently decline.

The effect of damping coefficient $\bar{\beta }$ on the self-oscillation is described in Figure 7, with parameters being set to ${\bar{x}}_{0}=0.5,\;{\bar{v}}_{0}=0,\;{\bar{C}}_{0}=0.3,\;\bar{k}=2,\;\bar{Q}=0.5,\;and\;\bar{a}=0.1.$ Figure 7a shows the cycles of self-oscillation relevant to different damping coefficient. A threshold damping coefficient$\;\bar{\beta }$ of approximately 0.044 exists in the phase transition between the static regime and self-oscillation regime. When $\bar{\beta }\geq 0.044,$ the energy dissipation due to damping exceeds the energy input, resulting in a static state. Conversely, self-oscillation occurs when $\bar{\beta }<0.044$. Figure 7a shows that the self-oscillation regime is observed at $\bar{\beta }=0.01$, $\bar{\beta }=0.02$, and $\bar{\beta }=0.03$. Further, Figure 7b presents the relationship between self-oscillation frequency and amplitude as functions of the damping coefficient $\bar{\beta }$. It is evident that an increase in the damping coefficient results in a decrease in both the f and A. As the damping coefficient rises, energy dissipation increases, leading to a further reduction in amplitude. Additionally, the velocity is also reduced, causing the sphere to take longer to complete a full oscillation cycle, which in turn corresponds to a lower frequency. This trend is consistent with previous research findings [30,35].

### 4.5. Influence of the Elastic Coefficient

**Figure 8:**
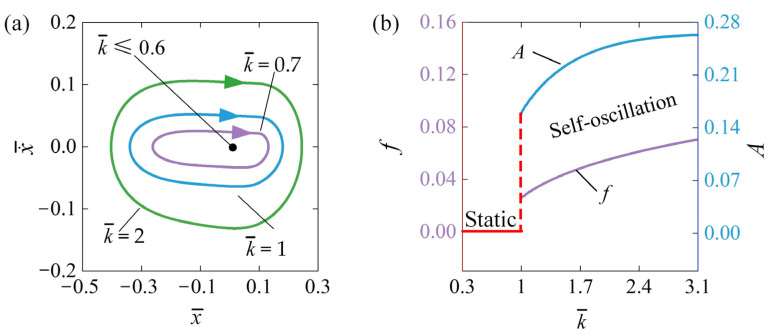
Influence of elastic coefficient $\bar{k}$ on self-oscillation for ${\bar{x}}_{0}=0.5,\;{\bar{v}}_{0}=0,\;\bar{\beta }=0.03,\;\bar{Q}=0.5,\;{\bar{C}}_{0}=0.3,\;and\;\bar{a}=0.1$. (**a**) Limit cycles; (**b**) effect of $\bar{k}$ on A and f. As the elastic coefficient rises, the self-oscillation A and f consistently increase.

Figure 8 describes the influence of elastic coefficient $\bar{k}$ on the self-oscillation. The parameters are set for ${\bar{x}}_{0}=0.5,\;{\bar{v}}_{0}=0,\;\bar{\beta }=0.03,\;\bar{Q}=0.5,\;{\bar{C}}_{0}=0.3,\;and\;\bar{a}=0.1.$ The limit cycles of self-oscillation associated with various values of the elastic coefficient are shown in Figure 8a. The threshold of the elastic coefficient $\bar{k}$ necessary to initiate self-oscillation is calculated to be approximately 0.6. If $\bar{k}\leq 0.6,\;$the oscillator remains static because energy input is insufficient to counteract the damping losses; consequently, the system ultimately settles into a static equilibrium position. However, self-oscillation can be activated if $\bar{k}>0.6$. Figure 8b further explores the relationship between f and A as functions of the elastic coefficient $\bar{k}$. It is clear that the self-oscillation f and A increase steadily with the rise in the elastic coefficient. This phenomenon occurs because materials with a larger elastic coefficient are more effective at harnessing thermal energy and transforming it into mechanical energy, thereby enhancing the system’s dynamic behavior.

### 4.6. Influence of the Junction Position

**Figure 9:**
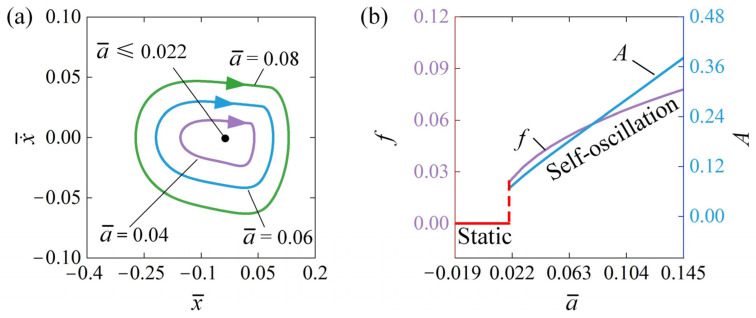
Influence of $\bar{a}$ on self-oscillation for the given values of ${\bar{x}}_{0}=0.5,\;{\bar{v}}_{0}=0,\;\bar{\beta }=0.03,\;\bar{k}=2,\;{\bar{C}}_{0}=0.3,\;and\;\bar{Q}=0.5$. (**a**) Limit cycles; (**b**) effect of $\bar{a}$ on A and f. With the increase in junction position, the self-oscillation A and f gradually increase.

Figure 9 describes the influence of the junction position $\bar{a}$ of the conductive and insulating segments on the self-oscillation for ${\bar{x}}_{0}=0.5,\;{\bar{v}}_{0}=0,\;\bar{\beta }=0.03,\;\bar{k}=2,\;{\bar{C}}_{0}=0.3,\;and\;\bar{Q}=0.5.$ Figure 9a illustrates the limit cycles associated with self-oscillation for various junction positions. A threshold junction position$\;\bar{a}$, approximately 0.022, is present during the transition from the static regime to the self-oscillation regime. When $\bar{a}\leq 0.022,\;$the system reaches a static state. In this condition, the energy supplied is insufficient to counterbalance the energy lost due to damping. As a result, the system settles into a stable equilibrium position and remains at rest. The system can enter a state of self-oscillation when the value of $\bar{a}$ exceeds 0.022. In Figure 9a, the limit cycles induced by self-oscillation are depicted for values of $\bar{a}=0.04$, $\bar{a}=0.06$, and $\bar{a}=0.08$. Meanwhile, Figure 9b illustrates how frequency and amplitude vary in relation to the junction position $\bar{a}$. Clearly, as the junction position increases, the self-oscillation f and A progressively grow. This phenomenon can be understood as follows: as the value of the junction position increases, it reduces the tensile force of the LCE fibers when the metal sphere moves along the conductive segment of the straight rod, allowing it to travel further to the right. This increase simultaneously creates more opportunities for the recovery of electrothermally induced contraction, which in turn reduces the tensile force as the metal sphere moves along the left side of the straight rod. This allows the metal sphere to travel further along the left side of the rod.

To summarize, this section provides a systematic analysis of how key dimensionless system parameters influence the amplitude A and frequency f of the self-oscillator, with the results being summarized in Table 3. The observed patterns of these influencing factors align with the conclusions of previous studies [44,48,79].

## 5. Conclusions

Self-oscillation is an elegant method that converts constant external stimuli into mechanical work, enabling sustained continuous motion and forming the basis for its widespread application in engineering. In this article, we propose a self-oscillator based on LCE operating under constant voltage. The system consists of an electrothermal-responsive LCE-LM composite fiber, a metal mass sphere, a straight rod with both conductive and insulating segments, conductive wire, and a direct current power supply. By employing the dynamic LCE model, we derive the governing equation for the motion of the oscillator. Numerical calculations reveal the existence of two distinct motion regimes: a static regime and a self-oscillation regime. It is demonstrated that self-oscillation arises from the electrothermal-responsive LCE-LM fiber contracting upon activation of the circuit and the continuous periodic motion sustained through the interplay between the metal mass sphere and a self-controlled dynamic circuit. Furthermore, the self-oscillation behavior is influenced by several system parameters, such as ${\bar{x}}_{0},\;{\bar{v}}_{0},\;\bar{\beta },\;\bar{k},\;{\bar{C}}_{0},\;\bar{a}$ and $\bar{Q}$. Our self-oscillator shows enhanced stability by minimizing the influence of gravity and other external disturbances. Furthermore, by substituting a straight rod with a curved one, we can achieve a curved trajectory for the mass sphere, leading to a more adaptable and easily controllable design. With these features in mind, a self-oscillator system utilizing LCE-LM fiber could be well suited for developing monitoring and alert devices, dynamic circuit systems, and for integrating actuators and controllers.

## Figures and Tables

**Table 1:** Material properties and geometric parameters.

Parameter	Definition	Value	Units
$C_{0}$	Thermal contraction coefficient	0~0.003	/℃
Q	Electrothermal flow	0~0.2	W
τ	Thermal relaxation time	0.05~0.15	s
$L_{0}$	Original length of the LCE-LM fiber	1~10	cm
k	Spring constant of the LCE-LM fiber	0~16	N/m
β	Damping coefficient	0~1	kg/s
m	Mass of the metal sphere	0~100	g
a	The junction position of the conductive and insulating segments	0~1	cm
$v_{0}$	Initial velocity	0~0.1	m/s
$x_{0}$	Initial displacement	0	cm
$k_{c}$	Heat transfer coefficient	0.05~0.1	W/℃
$T_{ext}$	Ambient temperature	0~20	℃

**Table 2:** Dimensionless parameters.

Parameter	${\bar{C}}_{0}$	$\bar{\beta }$	$\bar{k}$	${\bar{v}}_{0}$	$\bar{Q}$	$\bar{a}$	${\bar{x}}_{0}$
Value	0~0.5	0~0.3	0~8	0~0.1	0~2.5	0.1~0.25	0~1

**Table 3:** Effects of several key dimensionless parameters.

Dimensionless Parameter	Amplitude A	Frequency f
${\bar{x}}_{0}$	Remains constant with increasing ${\bar{x}}_{0}$	Remains constant with increasing ${\bar{x}}_{0}$
$\bar{Q}$	First increases and then decreases with increasing $\bar{Q}$	Increases with increasing $\bar{Q}$
${\bar{C}}_{0}$	Increases with increasing ${\bar{C}}_{0}$	Increases with increasing ${\bar{C}}_{0}$
$\bar{\beta }$	Decreases with increasing $\bar{\beta }$	Decreases with increasing $\bar{\beta }$
$\bar{k}$	Increases with increasing $\bar{k}$	Increases with increasing $\bar{k}$
$\bar{a}$	Increases with increasing $\bar{a}$	Increases with increasing $\bar{a}$

## Data Availability

The original contributions presented in this study are included in the article. Further inquiries can be directed to the corresponding author.

## References

[1] Jenkins A. (2013). Self-oscillation. Phys. Rep..

[2] Hu W., Lum G.Z., Mastrangeli M., Sitti M. (2018). Small-scale soft-bodied robot with multimodal locomotion. Nature.

[3] Iliuk I., Balthazar J.M., Tusset A.M., Piqueira J.R., Pontes D.B.R., Felix J.L., Bueno A.M. (2014). Application of passive control to energy harvester efficiency using a nonideal portal frame structural support system. J. Intell. Mater. Syst. Struct..

[4] Korner K., Kuenstler A.S., Hayward R.C., Audoly B., Bhattacharya K. (2020). A nonlinear beam model of photomotile structures. Proc. Natl. Acad. Sci. USA.

[5] Wehner M., Truby R.L., Fitzgerald D.J., Mosadegh B., Whitesides G.M., Lewis J.A., Wood R.J. (2016). An integrated design and fabrication strategy for entirely soft, autonomous robots. Nature.

[6] Cheng Y.C., Lu H.C., Lee X., Zeng H., Priimagi A. (2020). Kirigami-based light-induced shape-morphing and locomotion. Adv. Mater..

[7] Liao B., Zang H., Chen M., Wang Y., Lang X., Zhu N., Yang Z., Yi Y. (2020). Soft rod-climbing robot inspired by winding locomotion of snake. Soft Robot..

[8] Zhu Q., Liu W., Khoruzhenko O., Breu J., Hong W., Zheng Q., Wu Z. (2024). Animating hydrogel knotbots with topology-invoked self-regulation. Nat. Commun..

[9] Chun S., Pang C., Cho S.B. (2020). A micropillar-assisted versatile strategy for highly sensitive and efficient triboelectric energy generation under in-plane stimuli. Adv. Mater..

[10] Zhao D., Liu Y. (2020). A prototype for light-electric harvester based on light-sensitive liquid crystal elastomer cantilever. Energy.

[11] Preston D.J., Rothemund P., Jiang H.J., Nemitz M.P., Rawson J., Suo Z., Whitesides G.M. (2019). Digital logic for soft devices. Proc. Natl. Acad. Sci. USA.

[12] Rothemund P., Ainla A., Belding L., Preston D.J., Kurihara S., Suo Z., Whitesides G.M. (2018). A soft, bistable valve for autonomous control of soft actuators. Sci. Robot..

[13] El Helou C., Hyatt L.P., Buskohl P.R., Harne R.L. (2024). Intelligent electroactive material systems with self-adaptive mechanical memory and sequential logic. Proc. Natl. Acad. Sci. USA.

[14] Qi F., Li Y., Hong Y., Zhao Y., Qing H., Yin J. (2024). Defected twisted ring topology for autonomous periodic flip-spin-orbit soft robot. Proc. Natl. Acad. Sci. USA.

[15] Yoshida R. (2010). Self-oscillating gels driven by the Belousov-Zhabotinsky reaction as novel smart materials. Adv. Mater..

[16] Hua M., Kim C.S., Du Y., Wu D., Bai R., He X. (2021). Swaying gel: Chemo-mechanical self-oscillation based on dynamic buckling. Matter.

[17] Yang L.L., Chang L.F., Hu Y., Huang M.J., Ji Q.X., Lu P., Liu J.Q., Chen W., Wu Y.C. (2020). An autonomous soft actuator with light-driven self-sustained wavelike oscillation for phototactic self-locomotion and power generation. Adv. Funct. Mater..

[18] Ge F.j., Zhao Y. (2017). A new function for thermal phase transition-based polymer actuators: Autonomous motion on a surface of constant temperature. Chem. Sci..

[19] Hagiwara Y., Hasebe S., Fujisawa H., Morikawa J., Asahi T., Koshima H. (2023). Photothermally induced natural vibration for versatile and high-speed actuation of crystals. Nat. Commun..

[20] Wang Y., Liu J., Yang S. (2022). Multi-functional liquid crystal elastomer composites. Appl. Phys. Rev..

[21] Yang H., Zhang C., Chen B., Wang Z., Xu Y., Xiao R. (2023). Bioinspired design of stimuli-responsive artificial muscles with multiple actuation modes. Smart Mater. Struct..

[22] Xu T., Pei D., Yu S., Zhang X., Yi M., Li C. (2021). Design of MXene composites with biomimetic rapid and self-oscillating actuation under ambient circumstances. ACS Appl. Mater. Interfaces.

[23] Cunha M., Peeketi A.R., Ramgopal A., Annabattula R., Schenning A. (2020). Light-driven continual oscillatory rocking of a polymer film. ChemistryOpen.

[24] Zeng H., Lahikainen M., Liu L., Ahmed Z., Wani O.M., Wang M., Priimagi A. (2019). Light-fuelled freestyle self-oscillators. Nat. Commun..

[25] Hu Y., Ji Q., Huang M., Chang L., Zhang C., Wu G., Zi B., Bao N., Chen W., Wu Y. (2021). Light-driven self-oscillating actuators with phototactic locomotion based on black phosphorus heterostructure. Angew. Chem. Int. Ed..

[26] Sun J., Hu W., Zhang L., Lan R., Yang H., Yang D. (2021). Light-driven self-oscillating behavior of liquid-crystalline networks triggered by dynamic isomerization of molecular motors. Adv. Funct. Mater..

[27] Manna R.K., Shklyaev O.E., Balazs A.C. (2021). Chemical pumps and flexible sheets spontaneously form self-regulating oscillators in solution. Proc. Natl. Acad. Sci. USA.

[28] Li Z., Myung N.V., Yin Y. (2021). Light-powered soft steam engines for self-adaptive oscillation and biomimetic swimming. Sci. Robot..

[29] Wu H., Ge D., Chen J., Xu P., Li K. (2024). A light-fueled self-rolling unicycle with a liquid crystal elastomer rod engine. Chaos Solitons Fractals.

[30] Ge D., Dai Y., Liang H., Li K. (2024). Self-rolling and circling of a conical liquid crystal elastomer rod on a hot surface. Int. J. Mech. Sci..

[31] Guo K., Yang X., Zhou C., Li C. (2024). Self-regulated reversal deformation and locomotion of structurally homogenous hydrogels subjected to constant light illumination. Nat. Commun..

[32] Hu Z., Li Y., Lv J. (2021). Phototunable self-oscillating system driven by a self-winding fiber actuator. Nat. Commun..

[33] Zhao Y., Chi Y., Hong Y., Li Y., Yang S., Yin J. (2022). Twisting for soft intelligent autonomous robot in unstructured environments. Proc. Natl. Acad. Sci. USA.

[34] He Q., Wang Z., Wang Y., Wang Z., Li C., Annapooranan A.R., Zeng J., Chen R., Cai S. (2021). Electrospun liquid crystal elastomer microfiber actuator. Sci. Robot..

[35] Ge D., Liang H., Li K. (2024). Self-oscillation of a liquid crystal elastomer string-mass system under constant gradient temperature. J. Appl. Mech..

[36] Serak S.V., Tabiryan N.V., Vergara R., White T.J., Vaia R.A., Bunning T. (2010). Liquid crystalline polymer cantilever oscillators fueled by light. Soft Matter.

[37] Bai C., Kang J., Wang Y.Q. (2024). Light-induced motion of three-dimensional pendulum with liquid crystal elastomeric fiber. Int. J. Mech. Sci..

[38] Kuenstler A.S., Chen Y., Bui P., Kim H., DeSimone A., Jin L., Hayward R. (2020). Blueprinting photothermal shape-morphing of liquid crystal elastomers. Adv. Mater..

[39] Ge D., Li K. (2022). Self-oscillating buckling and postbuckling of a liquid crystal elastomer disk under steady illumination. Int. J. Mech. Sci..

[40] Gelebart G.A., Mulder D.J., Varga M., Konya A., Vantomme G., Meijer E.W., Selinger R.L.B., Broer D.J. (2017). Making waves in a photoactive polymer film. Nature.

[41] Shen B., Kang S.H. (2021). Designing self-oscillating matter. Matter.

[42] Zhao T., Fan Y., Lv J. (2022). Photomorphogenesis of diverse autonomous traveling waves in a monolithic soft artificial muscle. ACS Appl. Mater. Interfaces.

[43] Graeber G., Regulagadda K., Hodel P., Küttel C., Landolf D., Schutzius T., Poulikakos D. (2021). Leidenfrost droplet trampolining. Nat. Commun..

[44] Xu P., Sun X., Dai Y., Li K. (2024). Light-powered sustained chaotic jumping of a liquid crystal elastomer balloon. Int. J. Mech. Sci..

[45] Zhou X., Chen G., Jin B., Feng H., Chen Z., Fang M., Yang B., Xiao R., Xie T., Zheng N. (2024). Multimodal autonomous locomotion of liquid crystal elastomer soft robot. Adv. Sci..

[46] Ge D., Jin J., Dai Y., Xu P., Li K. (2022). Self-jumping of a liquid crystal elastomer balloon under steady illumination. Polymers.

[47] Hebner T.S., Korner K., Bowman C.N., Bhattacharya K., White T.J. (2023). Leaping liquid crystal elastomers. Sci. Adv..

[48] Qiu Y., Li K. (2024). Self-rotation-eversion of an anisotropic-friction-surface torus. Int. J. Mech. Sci..

[49] Li K., Qiu Y., Dai Y., Yu Y. (2024). Modeling the dynamic response of a light-powered self-rotating liquid crystal elastomer-based system. Int. J. Mech. Sci..

[50] Qiu Y., Ge D., Wu H., Li K., Xu P. (2024). Self-rotation of a liquid crystal elastomer rod under constant illumination. Int. J. Mech. Sci..

[51] Qiu Y., Dai Y., Li K. (2024). Self-spinning of liquid crystal elastomer tubes under constant light intensity. Commun. Nonlinear Sci. Numer. Simul..

[52] Yu Y., Zhou L., Du C., Zhu F., Dai Y., Ge D., Li K. (2024). Self-galloping of a liquid crystal elastomer catenary cable under a steady temperature field. Thin-Walled Struct..

[53] Sun X., Zhou K., Xu P. (2024). Chaotic self-beating of left ventricle modeled by liquid crystal elastomer. Thin-Walled Struct..

[54] Li K., Chen Z., Wang Z., Cai S. (2021). Self-sustained eversion or inversion of a thermally responsive torus. Phys. Rev. E.

[55] Ge D., Li K. (2022). Pulsating self-snapping of a liquid crystal elastomer bilayer spherical shell under steady illumination. Int. J. Mech. Sci..

[56] Vantomme G.A., Elands L.C.M., Gelebart G.A., Meijer E.W., Pogromsky A.Y., Nijmeijer H., Broer D.J. (2021). Coupled liquid crystalline oscillators in Huygens’ synchrony. Nat. Mater..

[57] Tong F., Kitagawa D., Bushnak I., Al-Kaysi R.O., Bardeen C.J. (2021). Light-powered autonomous flagella-like motion of molecular crystal microwires. Angew. Chem. Int. Ed..

[58] Li S., Bai H., Liu Z., Zhang X., Huang C., Wiesner L.W., Silberstein M., Shepherd R.F. (2021). Digital light processing of liquid crystal elastomers for self-sensing artificial muscles. Sci. Adv..

[59] Nie Z., Wang M., Huang S., Liu Z., Yang H. (2023). Multimodal self-sustainable autonomous locomotions of light-driven Seifert ribbon actuators based on liquid crystal elastomers. Angew. Chem. Int. Ed..

[60] Maeda S., Hara Y., Sakai T., Yoshida R., Hashimoto S. (2007). Self-walking gel. Adv. Mater..

[61] Li J., Mou L., Liu Z., Zhou X., Chen Y. (2022). Oscillating light engine realized by photothermal solvent evaporation. Nat. Commun..

[62] Dai L., Wang L., Chen B., Xu Z., Wang Z., Xiao R. (2023). Shape memory behaviors of 3D printed liquid crystal elastomers. Soft Sci..

[63] Wang L., Wei Z., Xu Z., Yu Q., Wu Z., Wang Z., Qian J., Xiao R. (2023). Shape morphing of 3D printed liquid crystal elastomer structures with precuts. ACS Appl. Polym. Mater..

[64] Chen B., Liu C., Xu Z., Wang Z., Xiao R. (2024). Modeling the thermo-responsive behaviors of polydomain and monodomain nematic liquid crystal elastomers. Mech. Mater..

[65] Nemati Y., Deng Z., Pi H., Guo H., Zhang H., Priimagi A., Zeng H. (2024). A scalable, incoherent-light-powered, omnidirectional self-oscillator. Adv. Intell. Syst..

[66] Wei Z., Wang P., Bai R. (2024). Thermomechanical coupling in polydomain liquid crystal elastomers. J. Appl. Mech..

[67] Kang J., Bai C., Liu S., Jia Y. (2024). Light-induced nontethered rolling of liquid crystal elastomer and carbon nanotube composite ring. ACS Appl. Polym. Mater..

[68] Wang Y., Yin R., Jin L., Liu M., Gao Y., Raney J., Yang S. (2023). 3D-printed photoresponsive liquid crystal elastomer composites for free-form actuation. Adv. Funct. Mater..

[69] Lu H., Zou Z., Wu X., Shi C., Xiao J. (2021). Fabrication and characterization of highly deformable artificial muscle fibers based on liquid crystal elastomers. J. Appl. Mech..

[70] Liu Y., Wu Y., Liang H., Xu H., Wei Y., Ji Y. (2023). Rewritable electrically controllable liquid crystal actuators. Adv. Funct. Mater..

[71] Zhang J., Guo Y., Hu W., Soon R.H., Davidson Z.S., Sitti M. (2021). Liquid crystal elastomer-based magnetic composite films for reconfigurable shape-morphing soft miniature machines. Adv. Mater..

[72] Sun Y., Wang L., Zhu Z., Li X., Sun H., Zhao Y., Peng C., Liu J., Zhang S., Li M. (2023). 3D-printed ferromagnetic liquid crystal elastomer with programmed dual-anisotropy and multi-responsiveness. Adv. Mater..

[73] Yakacki C.M., Saed M., Nair D.P., Gong T., Reed S.M., Bowman C.N. (2015). Tailorable and programmable liquid-crystalline elastomers using a two-stage thiol-acrylate reaction. RSC Adv..

[74] Wang M., Cheng Z., Zuo B., Chen X., Huang S., Yang H. (2020). Liquid crystal elastomer electric locomotives. ACS Macro Lett..

[75] Sun J., Wang Y., Liao W., Yang Z. (2021). Ultrafast, high-contractile electrothermal-driven liquid crystal elastomer fibers towards artificial muscles. Small.

[76] Liao W., Yang Z. (2022). The integration of sensing and actuating based on a simple design fiber actuator towards intelligent soft robots. Adv. Mater. Technol..

[77] Zhou L., Chen H., Li K. (2024). Optically-responsive liquid crystal elastomer thin film motors in linear/nonlinear optical fields. Thin-Walled Struct..

[78] Zhao J., Dai C., Dai Y., Wu J., Li K. (2024). Self-oscillation of cantilevered silicone oil paper sheet system driven by steam. Thin-Walled Struct..

[79] Liu J., Shi F., Song W., Dai Y., Li K. (2024). Modeling of self-oscillating flexible circuits based on liquid crystal elastomers. Int. J. Mech. Sci..

